# Stereoselective Solid-State Synthesis of Biologically Active Cyclobutane and Dicyclobutane Isomers via Conformation Blocking and Transference

**DOI:** 10.3390/molecules29122909

**Published:** 2024-06-19

**Authors:** Zhen Qin, Yunqiong Gu, Davidjames Young, Feilong Hu, Zhirong Luo

**Affiliations:** 1 Guangxi Key Laboratory of Chemistry and Engineering of Forest Products, Guangxi Minzu University, Nanning 530006, China; 2 College of Intelligent Metallurgy, Guangxi Modern Polytechnic College, Hechi 473000, China; 3 School of Environment and Life Science, Nanning Normal University, Nanning 530001, China; 4 Glasgow College UESTC, University of Electronic Science and Technology of China, Chengdu 611731, China; david.j.young@glasgow.ac.uk; 5 Guangxi Key Laboratory of Urban Water Environment, College of Chemistry & Environment Engineering, Baise University, Baise 533000, China

**Keywords:** solid state, photodimerization, isomers, anti-cancer

## Abstract

Conformations in the solid state are typically fixed during crystallization. Transference of “frozen” C=C conformations in 3,5-bis((E)-2-(pyridin-4-yl)vinyl)methylbenzene (CH_3_-3,5-bpeb) by photodimerization selectively yielded cyclobutane and dicyclobutane isomers, one of which (Isomer 2) exhibited excellent in vitro anti-cancer activity towards T-24, 7402, MGC803, HepG-2, and HeLa cells.

## 1. Introduction

**Scheme 1:**
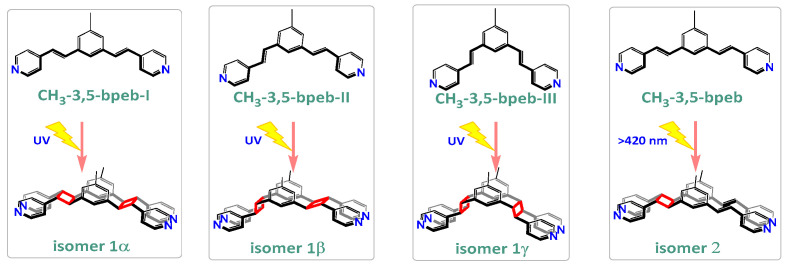
Conformers I, II, and III of 3,5-bis((E)-2-(pyridin-4-yl)vinyl)methylbenzene (CH_3_-3,5-bpeb) and four photodimerization products.

Conformational isomers involving rotation of carbon–carbon sigma bonds interconvert at room temperature due to typically low energy barriers (ca 12.6 KJ/mol) between staggered and eclipsed conformations [1]. The diene 3,5-bis((E)-2-(pyridin-4-yl)vinyl)-methylbenzene (CH_3_-3,5-bpeb) investigated in the present study exhibits a single, averaged ^1^H-NMR spectrum at room temperature in solution due to relatively rapid free rotation around the single bonds between pyridyl and vinyl groups. However, individual conformations are immobilized by self-assembly during crystallization, as determined by single-crystal X-ray diffraction. There are three planar, conjugated CH_3_-3,5-bpeb conformers, I, II, and III, in the solid state (Scheme 1). The photodimerization of each conformer would give an isomeric cyclobutane or dicyclobutane with the transfer of the solid-state conformational information to the cyclobutane configuration. This regio- and stereo-specificity of photodimerization usually rely on the constraints of the precursor molecules provided by metal coordination in a coordination polymer (CP) or metal organic framework (MOF) [2,3,4,5,6,7,8,9,10].

It is common for an organic reaction in solution to produce mixtures of isomers due to competing thermodynamic equilibria or kinetically controlled reactions [11]. The [2 + 2] photodimerization of alkenes usually yields a mixture of cyclobutane isomers [4,12,13,14,15,16], even in the solid state [17]. Molecular movement causes disorder in precursor orientations, increasing the likelihood of generating isomers [18,19]. Separation of these isomers is particularly challenging due to similarities in physical characteristics [13,20,21,22,23]. Crystalline solids are dominated by a myriad of intermolecular forces that restrict molecular movement and can lower the probability of generating isomeric mixtures [24,25]. The coordination geometries available in CPs and MOFs have been widely used to control the orientation of organic molecules in the solid state [26,27,28,29,30,31,32,33,34]. In this case, precursor molecules are “frozen” before the reaction to generate specific cycloisomers in photodimerization reactions [35]. In other words, the conformation of the olefinic bonds can be kinetically controlled by crystallization during the self-assembly of the CPs or MOFs. In addition, the kinetically arranged precursor can be successfully translated into thermodynamic photoproducts by solid-state photodimerization reactions.

## 2. Results and Discussion

**Scheme 2:**
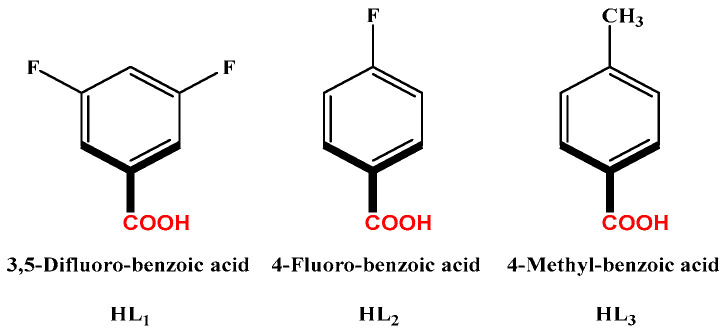
The carboxyl acid used in this work.

**Figure 1:**
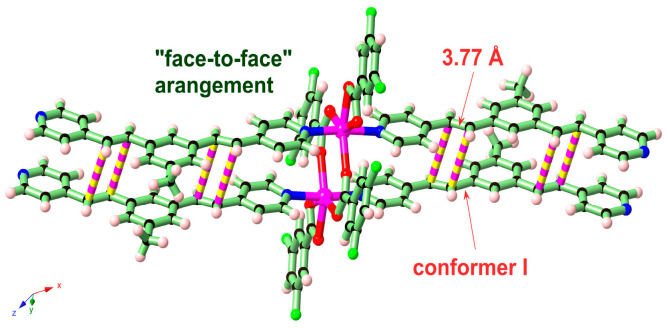
“Face-to-face” arrangement of CH_3_-3,5-bpeb pairs in CP_1_. CH_3_-3,5-bpeb ligands adopted conformation I exclusively. Color scheme: Cd, purple; C, black; O, red; N, blue; F, green, H, carnation.

**Figure 2:**
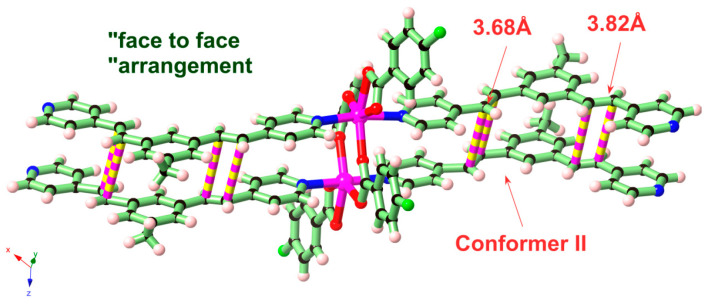
“Face-to-face” arrangement of CH_3_-3,5-bpeb pairs in CP_2_. CH_3_-3,5-bpeb ligands adopted conformer II exclusively. Color scheme: Cd, purple; C, black; O, red; N, blue; F, green, H, carnation.

**Scheme 3:**
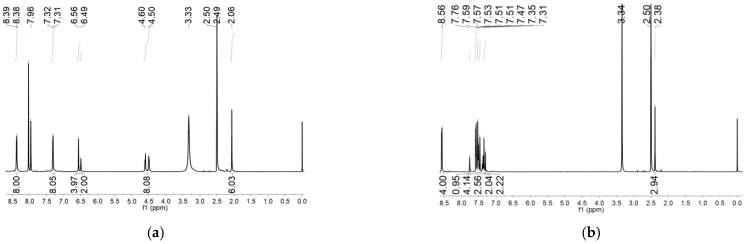
^1^H NMR spectra of (**a**) CP_1a,_ (**b**) CP_1_.

**Scheme 4:**
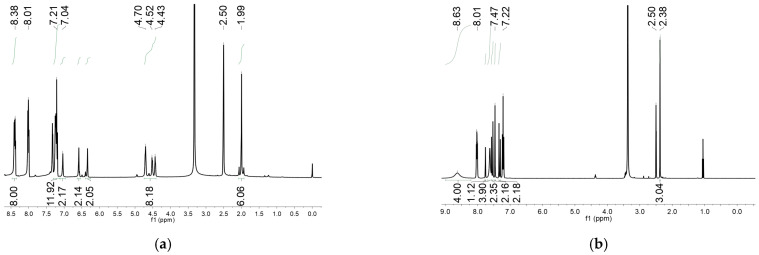
^1^H NMR spectra of (**a**) CP_2a_**_,_** (**b**) CP_2_.

**Figure 3:**
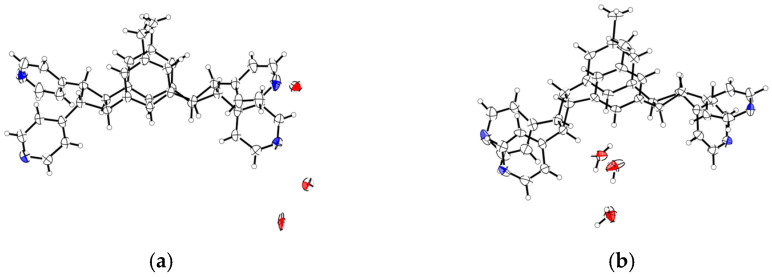
Crystal structures of (**a**) Isomer 1α and (**b**) Isomer 1β with 50% thermal ellipsoids.

**Figure 4:**
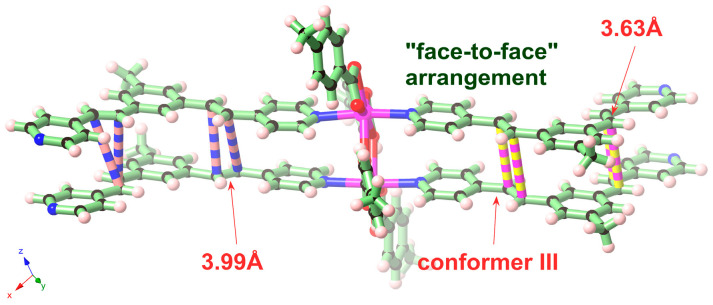
“Face-to-face” arrangement of CH_3_-3,5-bpeb pairs in CP_3_. CH_3_-3,5-bpeb ligands adopted both conformation III (shown) and conformation II. Color scheme: Cd, purple; C, black; O, red; N, blue; H, carnation.

**Scheme 5:**
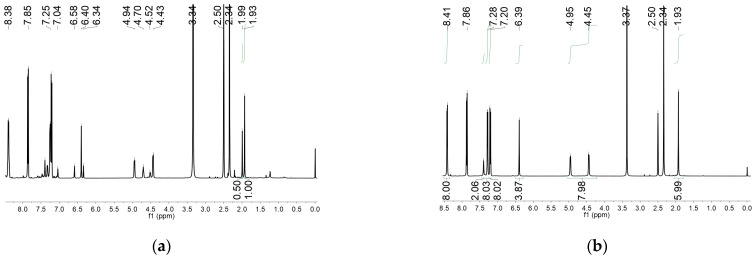
^1^H NMR spectrum of (**a**) CP_3a_ (273K)**_,_** (**b**) CP_3b_ (233K).

In this work, the functional molecule CH_3_-3,5-bpeb was employed as a bridging ligand to construct a series of 1D Cd(II)-based CPs by solvothermal reactions of 3CdSO_4_∙8H_2_O and various carboxylic acids (HL_1_-HL_3_) (Scheme 2). These 1D CPs underwent solid-state photodimerization to selectively afford pure cyclobutane isomers based on the preorganized conformation of the precursor molecules. The approach of “freezing” the original C=C conformation and “transferring” it through C-C coupling reaction makes it possible to selectively form specific isomers in a predictable manner. Ditopic ligand orientation in the solid state could be modulated with different carboxyl ligands (Scheme 2) [2,36]. The μ_2_-bridged carboxyl groups linked a pair of metal ions nearby at an appropriate distance [9,37]. In this way, the functional precursors were aligned in a “face-to-face” manner with the distance between C=C bonds within the Schmidt criteria for [2 + 2] cycloadditions [38]. The ditopic CH_3_-3,5-bpeb could potentially adopt conformers I, II, or III in the CPs (Scheme S1) [17]. The carboxyl ligands were instrumental in directing the arrangement of the bipyridine ligands. We observed that CH_3_-3,5-bpeb pairs adopted conformer I exclusively in the solid-state structure of CP_1_ and conformer II exclusively in CP_2_ (Figure 1 and Figure 2). Ultraviolet light irradiation of CP_1_ at room temperature produced only Isomer 1α (Scheme 3) and CP_2_ gave rise to pure Isomer 1β (Scheme 4) under the same conditions. It can be clearly found in the NMR spectrum of CP_1_ that the presence of 4.50 and 4.60 ppm can be attributed to cyclobutane peaks (Scheme 3). At the same time, the chemical shift at 7.35 and 7.47 ppm, which were assigned to a carbon–carbon double bond, disappeared after UV light irradiation (Scheme 3). Similarly, CP_2_ is accompanied by the disappearance of carbon–carbon double bonds and the formation of cyclobutane (4.43, 4.52, and 4.70 ppm) (Scheme 4). Obvious differences in chemical shift between the cyclobutane isomers were observed. Moreover, the easily identifiable chemical shifts of 2.06 and 1.99 ppm refer to the CH_3_- group of Isomer 1α and Isomer 1β, respectively, which are used to trace the reactants during the photodimerization process. The configurations of Isomer 1α (Figures S2 and S3) and Isomer 1β (Figures S4 and S5) were confirmed by mass spectrum and single crystal structure analysis (Figure 3). To some extent, once the conformation of the precursor is fixed in the CPs, the configuration of the photoproduct is predictable [39]. However, the conformation of CH_3_-3,5-bpeb in CP_3_ differed from those in CP_1_ and CP_2_. Single-crystal X-ray diffraction analysis revealed that both conformers II and III were present in CP_3_. The asymmetric unit contained a pair of stacked conformer III CH_3_-3,5-bpeb molecules and a pair of conformer II CH_3_-3,5-bpeb ligands face-to-face. Two pairs of criss-crossed C=C bonds were present (Figure 4); therefore, it is difficult to predict the configuration of the final photoproduct. Isomer 1γ should be formed from one pair of ligands in conformer III CH_3_-3,5-bpeb, but the photoproduct from the other pair of CH_3_-3,5-bpeb ligands could potentially lead to either of two isomers (Isomer 1α and Isomer 1β). Irradiation of CP_3_ at room temperature resulted in the formation of isomers 1β and 1γ in the ratio of 1/2 (Scheme 5). These results indicated that parallel preorganized C=C pairs reacted smoothly while the criss-crossed arranged C=C pair reacted more slowly (Scheme S2). The latter must move in a pedal motion prior to photodimerization. Molecules in a single crystal can undergo concerted reorganization in the solid state in response to external stimuli [40]. The porosity, thermal stability, and structural flexibility of CPs and MOFs permit limited control of molecular rotor dynamics [40,41]. Some of us have previously reported the pedal motion of ligands prior to photodimerization [42,43,44]. To some extent, control of movement at the molecular level can lead to control of the final product. Restricted movement increases the probability of producing a single cycloisomer. Temperature-dependent pedal motion occurs in different orientations (Scheme S3) and directs the configuration of the final structure. Low temperatures restrict this rotation of the C=C group and thereby influence the stereospecificity of the reaction. We therefore repeated the irradiation of CP_3_ at a lower temperature (−40 °C) and obtained Isomer 1γ as the only photoproduct (Scheme 5) with the appearance of the characteristic chemical shift of cyclobutane (4.45 and 4.95 ppm). The well-defined chemical shift at 1.93 ppm can be assigned to the methyl characteristic peak of Isomer 1γ. At 373 K, isomers 1α, 1β, and 1γ were formed in the ratio 0.19/0.43/1 (Figure S6). The higher temperature appears to accelerate the pedal motion, permitting the photocyclization to proceed. The results showed that the temperature-induced conformational change and the alteration of the alignment of the olefinic bonds of the CH_3_-3,5-bpeb ligands directed the stereospecificity of the final photoproducts. The pure Isomer 1γ (Figures S7 and S8) can be obtained by solid-state photodimerization of precursor CPs at low temperatures, which is difficult to synthesize through traditional methods [45,46,47].

A monocyclobutane compound was obtained when compounds CP_1_ and CP_2_ were irradiated under visible light (420 nm, cutoff) (Figure S9). That means only one C=C pair was dimerized to yield the intermediate monocyclobutane product which was confirmed using its NMR spectrum and Mass spectrum (Figures S10 and S11). The result showed that a higher light energy level (365 nm) was needed to initiate the other C=C bond pair, which has been reported recently [48].

The in vitro cytotoxicity of Isomers 1α, 1β, 1γ, and 2 against five cancer cell lines (MGC-803, T-24, HepG-2, BEL-7402, and HeLa) and one human normal cell line (HL-7702) were investigated using the MTT assay. All of the cyclobutane and dicyclobutane isomers exhibited an obvious inhibitory effect on these cancer cells. Isomer 2 displayed a higher anti-cancer activity against all cell lines relative to the other isomers (Table 1), with IC_50_ values of 7.0 ± 0.3, 6.2 ± 0.8, 8.9 ± 1.2, and 8.2 ± 0.9 μM against the T-24, HeLa, BEL-7402, and HepG-2 cell lines, respectively. The inhibitory effect of Isomer 2 was more obvious on tumor cells than the corresponding effect on a normal cell line (HL-7702).

**Figure 5:**
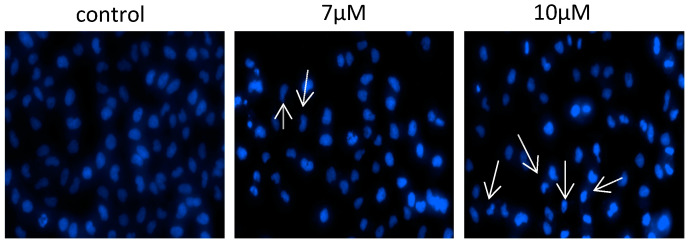
Assessment of nuclear morphological changes (white arrows) via Hoechst 33342 staining in T-24 cells after 24 h.

**Figure 6:**
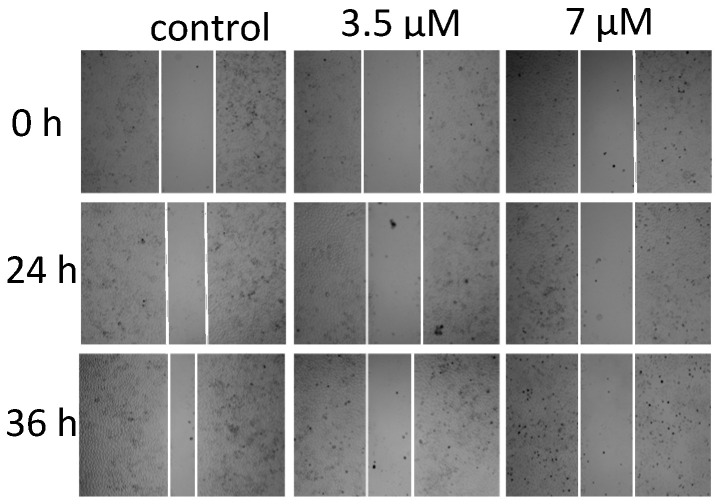
Effect of Isomer 2 on the in vitro migration potential of T-24 prostate cancer cells.

A Hoechst 33342 staining assay revealed that the Isomer 2 induced apoptosis in T-24 cells (Figure 5). Treatment with Isomer 2 also increased intracellular ROS (Figure S12) and calcium ion levels (Figure S13) and inhibited tubulin aggregation and cell migration in T-24 cells (Figure 6).

## 3. Materials and Methods

**Preparation of Cd-based coordination polymers:**

Preparation of [Cd(CH_3_-3,5-bpeb)(L_1_)_2_] (CP_1_): To a thick Pyrex tube was loaded 3CdSO_4_·8H_2_O (250 mg, 0.32 mmol), CH_3_-3,5-bpeb (11.7 mg, 0.024 mmol), HL_1_ (5.88 mg, 0.042 mmol), and 1.5 mL of DMF/H_2_O (*v*/*v* = 1:4) with one drop of concentrated HNO_3_. Tighten the cap and sonicate for 10 min. Then, the mixture was placed in an oven and programmed to 140 °C, maintaining the temperature for 12 h. After that, the reaction mixture was cooled to room temperature to form colorless crystals of CP_1_, which were collected by filtration, washed with EtOH and H_2_O, and dried in air. Yield 15.9 mg: (78% yield based on HL_1_). ^1^H NMR spectrum of CP_1_ (400 MHz, DMSO-*d*_6_) δ 8.57 (d, *J* = 6.0 Hz, 8H), 7.76 (s, 2H), 7.58 (d, *J* = 6.0 Hz, 8H), 7.53 (s, 4H), 7.49 (d, *J* = 15.6 Hz, 4H), 7.33 (d, *J* = 16.8 Hz, 4H), 2.38 (s, 6H).

Preparation of [Cd_2_(CH_3_-3,5-bpeb)_2_(L_2_)_4_] (CP_2_): Compound CP_2_ was synthesized in the same way as CP_1_, except with HL_2_ (5.9 mg, 0.042 mmol) instead of HL_1_. Yield 11.6 mg: (80% yield based on HL_2_). ^1^H NMR spectrum of CP_2_ (400 MHz, DMSO-*d*_6_) δ 8.63 (s, 8H), 8.02 (dd, *J* = 8.4, 6.0 Hz, 8H), 7.76 (s, 2H), 7.55 (d, *J* = 16.4 Hz, 4H), 7.47 (s, 4H), 7.32 (d, *J* = 16.4 Hz, 4H), 2.38 (s, 6H).

Preparation of [Cd_4_(CH_3_-3,5-bpeb)_4_(L_3_)_8_](H_2_O) (CP_3_): Compound CP_3_ was synthesized in the same way as CP_1_, except with HL_3_ (5.7 mg, 0.042 mmol) instead of HL_1_. Yield 12.3 mg: (86% yield based on HL_3_). ^1^H NMR spectrum of CP_3_ (400 MHz, DMSO-*d*_6_) δ 8.60 (d, *J* = 6.0 Hz, 8H), 7.76 (s, 2H), 7.61 (d, *J* = 6.0 Hz, 8H), 7.55 (d, *J* = 16.4 Hz, 4H), 7.46 (s, 4H), 7.33 (d, *J* = 16.4 Hz, 4H), 2.38 (s, 6H).

Photo-irradiation: Single crystals of compounds CP_1_–CP_3_ were placed in a long glass tube and irradiated with an LED lamp (100 W, 365 nm or >420 nm, cut-off) for a period of time to form the photoproducts CP_1a_–CP_3a_.

Preparation of CP_1a_: The CP_1_ crystal was irradiated for 7 h, finally obtaining the dark brown CP_1a_. Yield 99% (based on CP_1_). ^1^H-NMR spectrum of CP_1a_ (400 MHz, DMSO-*d*_6_ ppm, after UV irradiation): *δ* 8.39 (d, *J* = 4.0 Hz 8H), 7.52 (d, *J* = 6.8 Hz 8H), 6.56 (s, 4H), 6.49 (s, 2H), 4.60 (d, *J* = 6.4 Hz 4H), 4.50 (d, *J* = 6.4 Hz 4H), 2.06 (s, 6H).

Preparation of CP_1_′: The CP_1_ crystal was irradiated (>420 nm) for 5 h, finally obtaining the yellow CP_1_′. Yield 95% (based on CP_1_). ^1^H-NMR spectrum of CP_1_′ (400 MHz, DMSO-*d_6_* ppm, after UV irradiation): *δ* 8.48 (d, *J* = 6.0 Hz 4H), 8.38 (d, *J* = 6.0 Hz 4H), 7.76 (s, 1H), 7.55 (d, *J* = 16.4 Hz 2H), 7.39 (s, 1H), 7.32 (d, *J* = 16.4 Hz 2H), 7.29 (d, *J* = 6.0 Hz 2H), 7.14 (d, *J* = 6.0 Hz 2H), 7.07 (s, 1H), 4.71 (d, *J* = 6.8 Hz 1H), 4.57 (d, *J* = 6.4 Hz 1H), 2.20 (s, 3H).

Preparation of CP_2a_: The CP_2_ crystal was irradiated for 6 h, finally obtaining the dark brown CP_2a_. Yield 95% (based on CP_2_). ^1^H-NMR spectrum of CP_2a_ (400 MHz, DMSO-*d_6_* ppm, after UV irradiation): *δ* 8.39 (dd, *J* = 11.6 Hz, *J* = 5.6 Hz 8H), 7.33(d, *J* = 4.8 Hz 4H), 7.23 (d, *J* = 5.6 Hz 4H), 7.04 (s, 2H), 6.59 (s, 2H), 6.34 (s, 2H), 4.70–4.43 (m, 8H), 1.99 (s, 6H).

Preparation of CP_3a_: The CP_3_ crystal was irradiated for 6 h at 273 K, finally obtaining the brown CP_3a_. Yield 67% (based on CP_3_). At 233 K, finally obtained the pale yellow CP_3a_. Yield 100% (based on CP_3_). At 373 K, finally obtained the dark brown CP_3a_. Yield 61% (based on CP_3_). ^1^H-NMR spectrum of CP_3a_ (400 MHz, DMSO-*d*_6_ ppm, after UV irradiation): *δ* 8.42 (d, *J* = 6.0 Hz 8H), 7.39 (s, 2H), 7.28 (d, *J* = 6.0 Hz 8H), 6.39 (s, 4H), 4.96 (d, *J* = 6.0 Hz 4H), 4.44 (d, *J* = 6.0 Hz 4H), 1.93 (s, 6H).

Preparation of Isomer 1α: Na_2_Y·2H_2_O (Y = ethylenediamine tetraacetate) (560 mg), CP_1_ (387 mg), H_2_O (20 mL), and CH_2_Cl_2_ (30 mL) were mixed in a 100 mL flask, Then, NaOH solution (1.0 mol·L^−1^, 3.0 mL) was slowly added dropwise to the mixture, which was stirred for about 4 h. After the reaction was completed, extraction was repeated three times with CH_2_Cl_2_ (3 × 30 mL), and the target product was separated from the mixture. The organic phases were combined together and the combined organic layer was dried over anhydrous Na_2_SO_4_. Isomer 1α was finally obtained as a white powder. Yield: 84 mg (65% based on CP_1_). ^1^H-NMR spectrum of Isomer 1α (400 MHz, CDCl_3_ ppm): δ 8.44 (d, *J* = 5.7 Hz, 8H), 7.12 (d, *J* = 6.0 Hz, 8H), 6.54 (s, 4H), 6.32 (s, 2H), 4.45 (d, *J* = 12.6 Hz, 8H), 2.13 (s, 6H). HRMS Calcd for (C_42_H_36_N_4_ + H^+^): 597.2940; found: 597.2990.

Preparation of Isomer 1β: Na_2_Y·2H_2_O (Y = ethylenediamine tetraacetate) (560 mg), CP_2_ (276 mg), H_2_O (20 mL), and CH_2_Cl_2_ (30 mL) were mixed in a 100 mL flask, Then, NaOH solution (1.0 mol·L^−1^, 3.0 mL) was slowly added dropwise to the mixture, and the reaction was stirred for about 6 h. After the reaction was completed, extraction was repeated three times with CH_2_Cl_2_ (3 × 30 mL), and the target product was separated from the mixture. The organic phases were combined together and the combined organic layer was dried over anhydrous Na_2_SO_4_. The Isomer 1β was finally obtained as a white powder. Yield: 106.9 mg (76% based on CP_2_). ^1^H-NMR spectrum of Isomer 1β (400 MHz, CDCl_3_ ppm): δ 8.43 (d, *J* = 10.8 Hz, 8H), 7.13 (s, 4H), 7.04 (s, 4H), 6.74 (s, 2H), 6.57 (s, 2H), 6.35 (s, 2H), 4.30–4.57 (m, 8H), 2.07 (s, 6H). HRMS Calcd for (C_42_H_36_N_4_ + H^+^): 597.2940; found: 597.3012.

Preparation of Isomer 1γ: Na_2_Y·2H_2_O (Y = ethylenediamine tetraacetate) (560 mg), CP_3_ (289 mg), H_2_O (20 mL), and CH_2_Cl_2_ (30 mL) were mixed in a 100 mL flask, Then, NaOH solution (1.0 mol·L^−1^, 3.0 mL) was slowly added dropwise to the mixture, and the reaction was stirred for about 2 h. After the reaction was completed, extraction was repeated three times with CH_2_Cl_2_ (3 × 30 mL), and the target product was separated from the mixture. The organic phases were combined together and the combined organic layer was dried over anhydrous Na_2_SO_4_. After removal of the solvent (CH_2_Cl_2_) under reduced pressure, Isomer 1γ was finally obtained as a white powder. Yield: 111.9 mg (81% based on CP_3_). ^1^H-NMR spectrum of Isomer 1γ (400 MHz, CDCl_3_ ppm): δ 8.45 (d, *J* = 5.6 Hz, 8H), 7.12 (d, *J* = 6.0 Hz, 8H), 6.54 (s, 4H), 6.32 (s, 2H), 4.64 (d, *J* = 5.2 Hz, 4H), 4.26 (d, *J* = 5.6 Hz, 4H), 2.13 (s, 6H). HRMS Calcd for (C_42_H_36_N_4_ + H^+^): 597.2940; found: 597.2995.

Preparation of Isomer 2: Na_2_Y·2H_2_O (Y = ethylenediamine tetraacetate) (560 mg), CP_1_′ (CP_1_ after UV irradiation >420 nm) (297 mg), H_2_O (20 mL), and CH_2_Cl_2_ (30 mL) were mixed in a 100 mL flask, then NaOH solution (1.0 mol·L^−1^, 3.0 mL) was slowly added dropwise to the mixture, which was stirred for about 8 h. After the reaction was completed, extraction was repeated three times with CH_2_Cl_2_ (3 × 30 mL), the organic phases were combined together, and the combined organic layer was dried over Na_2_SO_4_. After removal of the solvent under reduced pressure, the residue was purified by column chromatography on silica. Isomer 2 was finally obtained as a white powder. Yield: 45.2 mg (35% based on CP_1_′). ^1^H-NMR spectrum of Isomer 2 (400 MHz, CDCl_3_ ppm): δ 8.52 (d, *J* = 5.6 Hz, 4H), 8.42 (d, *J* = 5.6 Hz, 4H), 7.25 (d, *J* = 5.6 Hz, 4H), 7.15 (d, *J* = 16.0 Hz, 2H), 7.09 (s, 2H), 7.05 (d, *J* = 5.6 Hz, 4H), 6.88 (s, 2H), 6.79 (d, *J* = 16.4 Hz, 2H), 4.48 (d, *J* = 16.8 Hz, 4H), 2.26 (s, 6H). HRMS Calcd for (C_42_H_36_N_4_ + H^+^): 597.2940; found: 597.2984.

Measurement of Ca^2+^ generation: Ca^2+^ is an important part of life activities. It is often used as a signal molecule for the death of cells. When cells are stimulated by specific signals during life activities, the calcium channels in the cells are opened, resulting in an increase in intracellular calcium concentration. At this time, the fluorescence microscope was used to determine the Ca^2+^ level of the cells stained with the Fluo-3 AM staining reagent. The main reason is that the stained kit can pass through the cell membrane and be cut into Fluo-3 by esterase. Fluo-3 can combine with calcium to produce strong green fluorescence. As shown in Figure S13, after treatment with Isomer 2 (0, 7, and 10 μM), the green fluorescence intensity increased significantly in T-24 cells. Hence, Isomer 2 can increase the intracellular levels of Ca^2+^.

Hoechst 33342 nucleic acid staining: Hoechst 33342 staining is a fluorescent dye that is firmly bound to the nucleus. It will be accompanied by nuclear damage or chromatin condensation in the process of apoptosis. As nuclear shrinkage is one of the most important features of apoptosis, Hoechst 33342 will wither dead cells, which are then stained with bright colors. Therefore, it is very important to detect the nuclear damage or chromatin concentration of T-24 cancer cells after Isomer 2 treatment. The Hoechst 33342 staining technique is performed according to the methods in the literature. The results of Figure 5 after treatment with Isomer 2 (0, 7, and 10 μM) show that the nuclear structure of the untreated cells was intact, while cells treated with Isomer 2 showed nuclear shrinkage or fragmentation. The fluorescence intensity increased significantly.

Intracellular ROS (Reactive Oxygen Species): Usually, people use DCFH-DA (2′,7′-dichlorofluorescein diacetate) probe to monitor the ROS content in cells. Although the probe itself does not have the property of directly generating fluorescence, DCFH-DA can freely pass through the cell membrane. After entering the cell, DCFH-DA can be hydrolyzed by esterase to DCFH. The hydrolyzed DCFH will not penetrate the cell membrane, resulting in no accumulation in the cell. Non-fluorescent DCFH can be oxidized by ROS in cells to produce DCF (2′,7′-Dichlorofluorescein) with green fluorescence. At the same time, the green fluorescence intensity is directly proportional to the ROS level. Therefore, the intensity of green fluorescence in cells can basically reflect the concentration of ROS in cells. As shown in Figure S12, the green fluorescence in T-24 cells was enhanced after 18 h of treatment with Isomer 2 (0 and 7 μM) compared to the untreated control. Therefore, Isomer 2 can increase ROS levels in T-24 cells.

Effect of Isomer 2 on tumor cell migration: After the T-24 cells adhered to the wall, we used a pipette tip to “scratch” the cell plate. Using DMSO as a negative control, we added Isomer 2 (3.5 μM and 7 μM) to treat the cells, and processed the photo record at different times. The results are shown in Figure 6. The scratch width of the DMSO control group became significantly narrower with time and the closure rate of the scratches in the Isomer 2 treatment group was significantly slower. In addition, the effect was more obvious with increasing concentrations. The results show that Isomer 2 can inhibit the migration ability of T-24 cells. 

Effect of Isomer 2 on in vitro migration potential of T-24 prostatecancer cells. Scratches were created with a sterile 200 mL pipette and images were captured using fluorescence microscopy (Cytation 5 Cell Imaging Multi-Mode Reader, BioTek Instruments, Inc., Winooski, VT, USA) at 0 h, 24 h, and 36 h after treatment with 0, 3.5 μM, and 7 μM of Isomer 2, respectively.

## 4. Conclusions

In summary, we have prepared four new cyclobutane and dicyclobutane isomers through stereoselective solid-state photodimerization of the corresponding ditopic dipyridyl alkene ligands. Isomer formation was determined by the orientation of the C=C bonds in the solid state as dictated by the coordination environment under the influence of the dicarboxylate ligands. Temperature also played a role, with lower temperatures restricting molecular motion and improving selectivity. Manipulating unfavorable thermodynamic motion is a general strategy for controlling the stereochemistry of the final photoproduct. These results are in striking contrast to the corresponding photodimerization reactions in solution and the simplified isolation of isomerically pure products. Isomer 2 showed excellent antitumor activity toward T-24 HeLa and BEL-7402 cell lines, with IC_50_ values in the μM range.

## Figures and Tables

**Table 1:** IC_50_ (μM) values for Isomer 1α, 1β, 1γ, and Isomer 2 against different cell lines.

Compounds	MGC-803	T-24	HepG-2	BEL-7402	HeLa	HL-7702
Isomer 1α	>20	>20	>20	>20	>20	>20
Isomer 1β	7.6 ± 0.2	7.3 ± 0.5	10.7 ± 0.6	18.8 ± 0.6	6.3 ± 0.3	>20
Isomer 1γ	>20	8.2 ± 0.9	10.6 ± 0.3	10.8 ± 0.7	13.3 ± 1.3	>20
Isomer 2	>20	7.0 ± 0.3	8.2 ± 0.9	8.9 ± 1.2	6.2 ± 0.8	>20

## Data Availability

The original contributions presented in this study are included in this article. Further inquiries can be directed to the corresponding author.

## Supplementary Materials

The following supporting information can be downloaded at: https://www.mdpi.com/article/10.3390/molecules29122909/s1. Table S1: Summary of crystal data and structure refinement parameters for compounds **CP_1_**, **CP_2_**, **CP_3_**, **Isomer 1α** and **Isomer 1β**; Scheme S1: The offset and face to face arrangements and possible conformers of **CH_3_-3,5-bpeb**; Figure S1: ^1^H NMR spectrum of **CH_3_-3,5-bpeb** (*d*_6_-DMSO); Figure S2: The ^1^H (a), ^13^C (b), H-H COSY (c), HSQC (d), HMBC (e) and NOESY (f) NMR spectra of Isomer 1α in CDCl_3_; Figure S3: The positive-ion ESI mass spectrum of **Isomer 1α**; Figure S4: The ^1^H (a), ^13^C (b), H-H COSY (c), HSQC (d), HMBC (e) and NOESY (f) NMR spectra of **Isomer 1β** in CDCl_3_; Figure S5: The positive-ion ESI mass spectrum of **Isomer 1β**; Scheme S2: Photodimerization between the parallel arranged C=C with the crisscross aligned C=C unreacted; Scheme S3: Representation of the pedal motion in adjacent CH_3_-3,5-bpeb molecules; Figure S6: ^1^H NMR spectrum of **CP_3c_** (*d*_6_-DMSO, after UV irradiation at 373K); Figure S7: The ^1^H (a), ^13^C (b), H-H COSY (c), HSQC (d), HMBC (e) and NOESY (f) NMR spectra of **Isomer 1γ** in CDCl_3_; Figure S8: The positive-ion ESI mass spectrum of **Isomer 1γ**; Figure S9: ^1^H NMR spectrum **CP_3_′** (*d*_6_-DMSO **CP_3_** after irradiation > 420 nm); Figure S10: The ^1^H (a), ^13^C (b), H-H COSY (c), HSQC (d), HMBC (e) and NOESY (f) NMR spectra of **Isomer 2** in CDCl_3_; Figure S11: The positive-ion ESI mass spectrum of **Isomer 2**; Figure S12: Changes of ROS on T-24 cells treated with **Isomer 2**; Figure S13: Changes in Ca^2+^ concentration in T-24 cells treated with **Isomer 2**. Refs. [49,50] are cited in the Supplementary Materials.
