# Supplementary material for: Stereoselective Solid-State Synthesis of Biologically Active Cyclobutane and Dicyclobutane Isomers via Conformation Blocking and Transference
Source: Molecules. 2024 Jun 19;29(12):2909. doi: 10.3390/molecules29122909 (PMC11206361; doi:10.3390/molecules29122909)
Supplement: Supplementary file 1 [file molecules-29-02909-s001.zip › molecules-3007997-supplementary.pdf]

Supporting Information

Stereoselective Solid-state Synthesis of Biologically Active Cyclobutane and Dicyclobutane Isomers *via* Conformation Blocking and Transference

Zhen Qin[1,4‡], Yun-Qiong Gu[2‡], David James Young [5], Fei-Long Hu[1,*] and Zhi-Rong Luo[3,*]

1 Guangxi Key Laboratory of Chemistry and Engineering of Forest Products, Guangxi Minzu University; Nanning 530006, China
2 School of Environment and Life science, Nanning Normal University; Nanning 530001, China
3 Guangxi Key Laboratory of Urban Water Environment, College of Chemistry & Environment Engineering, Baise University; Baise 533000, China
4 College of Intelligent Metallurgy, Guangxi Modern Polytechnic College; Hechi 473000, China
5 Glasgow College UESTC, University of Electronic Science and Technology of China; Chengdu 611731,China

Table of contents

General methods 3

Single crystal X-ray diffraction analysis 3

Table S1.Summary of crystal data and structure refinement parameters for compounds $\mathbf{CP_1}$**,** $\mathbf{CP_2}$, $\mathbf{CP_3}$, **Isomer 1α** and **Isomer 1β**. 4

Scheme S1.The offset and face to face arrangements and possible conformers of $\mathbf{CH_3}$**-3,5-bpeb**. 4

Figure S1. ^{1}H NMR spectrum of $\mathbf{CH_3}$**-3,5-bpeb** (*d*$_6$-DMSO). 5

Figure S2. The ^{1}H (a), ^{13}C (b), H-H COSY (c), HSQC (d), HMBC (e) and NOESY (f) NMR spectra of **Isomer 1α** in $CDCl_3$. 8

Figure S3. The positive-ion ESI mass spectrum of **Isomer 1α**. 8

Figure S4. The ^{1}H (a), ^{13}C (b), H-H COSY (c), HSQC (d), HMBC (e) and NOESY (f) NMR spectra of **Isomer 1β** in $CDCl_3$. 11

Figure S5. The positive-ion ESI mass spectrum of **Isomer 1β**. 12

Scheme S2. Photodimerization between the parallel arranged C=C with the crisscross aligned C=C unreacted. 12

Scheme S3. Representation of the pedal motion in adjacent CH_3-3,5-bpeb molecules. 13

Figure S6. ^{1}H NMR spectrum of $\mathbf{CP_{3c}}$ (*d*$_6$-DMSO, after UV irradiation at 373K) 13

Figure S7. The ^{1}H (a), ^{13}C (b), H-H COSY (c), HSQC (d), HMBC (e) and NOESY (f) NMR spectra of **Isomer 1γ** in $CDCl_3$. 16

Figure S8. The positive-ion ESI mass spectrum of **Isomer 1γ**. 17

Figure S9. ^{1}H NMR spectrum $\mathbf{CP_3}$' (*d*$_6$-DMSO $\mathbf{CP_3}$ after irradiation >420 nm). 18

Figure S10. The ^{1}H (a), ^{13}C (b), H-H COSY (c), HSQC (d), HMBC (e) and NOESY (f) NMR spectra of **Isomer 2** in $CDCl_3$. 21

Figure S11. The positive-ion ESI mass spectrum of **Isomer 2**. 21

Figure S12. Changes of ROS on T-24 cells treated with **Isomer 2**. 22

Figure S13. Changes in Ca^{2+} concentration in T-24 cells treated with **Isomer 2**. 22

References 23

Experimental

General methods

Synthesis

3,5-bis((E)-2-(pyridin-4-yl)vinyl)Methylbenzene (CH_3-3,5-bpeb), In a thick Pyrex tube was loaded 1,3-dibromo-5-methylbenzene (2.50 g, 1.0 mol), 4-vinylpyridine (2.20 g, 2.1 mol), and 40 mL of DMF with 1% Pd. Fill with inert gas nitrogen protection. The tube was sealed and heated at 100 °C for 2 h. After that, it was cooled to room temperature to form CH_3-3,5-bpeb(Fig. S1), which were collected by filtration, washed with H_2O, and dried in air. Yield: 2.84 g (95.3% based on 1,3-dibromo-5-methylbenzene) [1]. All chemicals used here were of analytical grade quality and obtained commercially and used without further purification. Deionized water (distilled) was used throughout the experiments. The ^{1}H NMR spectra were recorded at ambient temperature on a Bruker AVANCE 400M or 600M spectrometer ($CDCl_3$ or DMSO-d_6). Chemical shifts are reported in parts per million (ppm) and referenced with TMS for ^{1}H NMR. ^{13}C NMR spectra were recorded at a resonance frequency of 101.6 MHz on a BRUKER ADVANCE 400 MHz spectrometer at ambient temperature. High-resolution mass spectra (HRMS) were obtained by electrospray ionization (ESI). The photo-irradiation experiments were conducted with a high-pressure mercury lamp.

Single crystal X-ray diffraction analysis

Single crystal X-ray data for **CP_1, CP_2, CP_3, Isomer 1α, Isomer 1β** were collected on a Bruker Apex II X-ray diffractomete equipped with Mo Kα radiation (λ=0.71073 Å). The integrated intensity data for each reflection was collected by reduction of the data frames with the program Apex II. Hydrogen atoms were located in perfect positions and set riding on individual parent atoms. The structures were solved with the program *SHELXS-2018* and refined by full-matrix least squares on F^2 using the program *SHELXTL-2018*[2]. All non-hydrogen atoms were refined with anisotropic thermal parameters. The parameters used intensity collection and refinements of **CP_1, CP_2, CP_3, Isomer 1α, Isomer 1β** are summarized in Table S1. The CCDC code of 1982013-1982017 are for **CP_1, CP_2, CP_3, Isomer 1α, Isomer 1β** respectively.

Table S1.Summary of crystal data and structure refinement parameters for compounds **CP_1, CP_2, CP_3, Isomer 1α** and **Isomer 1β**.

	CP_1	CP_2	CP_3	Isomer 1α	Isomer 1β
Emperical formula	$C_{35}H_{24}Cd_1F_4N_2O_4$	$C_{70}H_{52}F_4Cd_2N_4O_8$	$C_{148}H_{130}Cd_4N_8O_{17}$	$C_{84}H_{72}N_8O_8$	$C_{42}H_{42}N_4O_3$
Formula weight	724.97	1377.98	2742.25	1321.50	650.29
Crystal system	monoclinic	monoclinic	monoclinic	monoclinic	monoclinic
Space group	*C2/c*	*C2/c*	*P2/n*	*P2/c*	*P21/c*
a/Å	32.969(9)	21.8105(13)	10.9746(8)	11.6161(2)	10.1778(12)
b/Å	12.530(3)	12.6807(8)	27.119(2)	11.6161(2)	20.568(2)
c/Å	34.790(9)	27.2770(14)	22.7457(17)	46.0453(12)	16.6888(18)
α/°	90.000	90.000	90.000	90	90.000
β/°	152.777(9)	124.735(3)	103.370(2)	90	95.138(3)
γ/°	90.000	90.000	90.000	120.00	90.000
V/Å^3	6574.5(4)	6199.7(7)	6586.1(8)	5416.7(14)	3479.6(7)
D_c/g cm^{-3}	1.465	1.476	1.383	1.224	1.242
Z	8	4	2	3	4
μ (Mo-Kα)/mm^{-1}	0.727	0.758	0.705	0.636	0.079
Total reflections	81215	30527	43531	41806	36567
Unique reflections	9865	9116	7314	6838	6127
No. observations	4521	4563	3081	3143	3747
No. parameters	416	822	804	813	450
F(000)	2912.0	2784.8	2798.63	2088.0	1384.0
$R_1{}^a$	0.0382	0.0511	0.0596	0.0543	0.0635
$wR_2{}^b$	0.1239	0.1263	0.1525	0.1675	0.1697
GOF^c	1.043	1.150	1.125	1.182	1.027

$^aR_1=\Sigma||F_0|-|F_c||/\Sigma|F_0|$. $^bwR_2=\{\Sigma w(F_0{}^2-F_c{}^2)^2/\Sigma w(F_0{}^2)^2\}^{1/2}$. ^{c}GOF$=\{\Sigma w((F_0{}^2-F_c{}^2)^2)/(n-p)\}^{1/2}$, where n=number of reflections and p=total number of parameters refined.

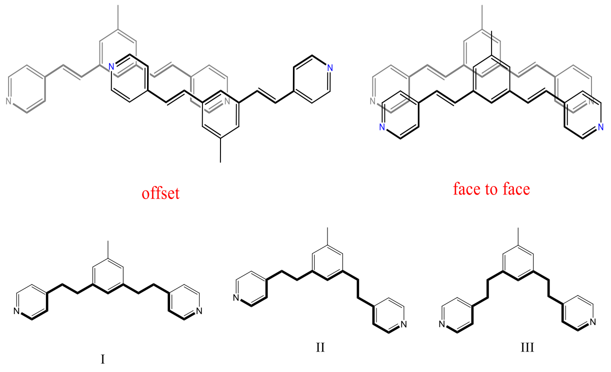

Scheme S1.The offset and face to face arrangements and possible conformers of **CH_3-3,5-bpeb**.

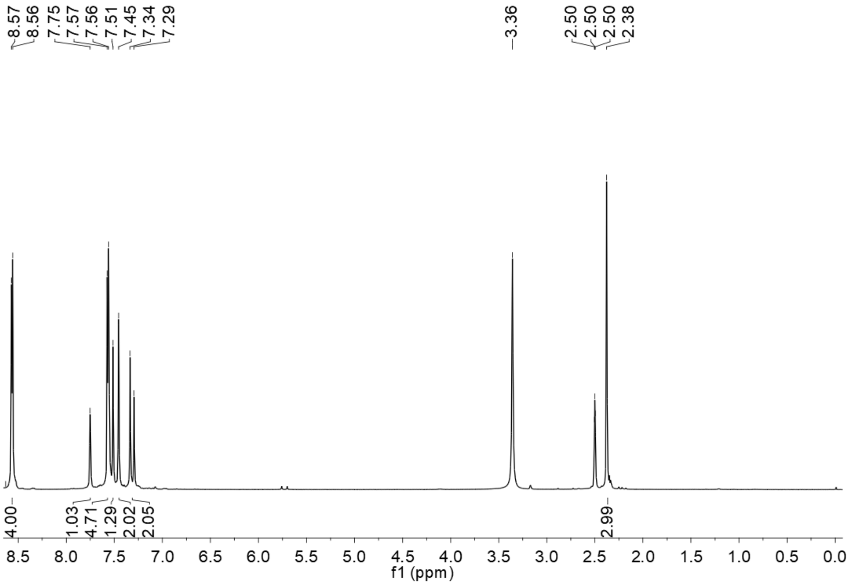

Figure S1. ^{1}H NMR spectrum of **CH3-3,5-bpeb** (d_6-DMSO).

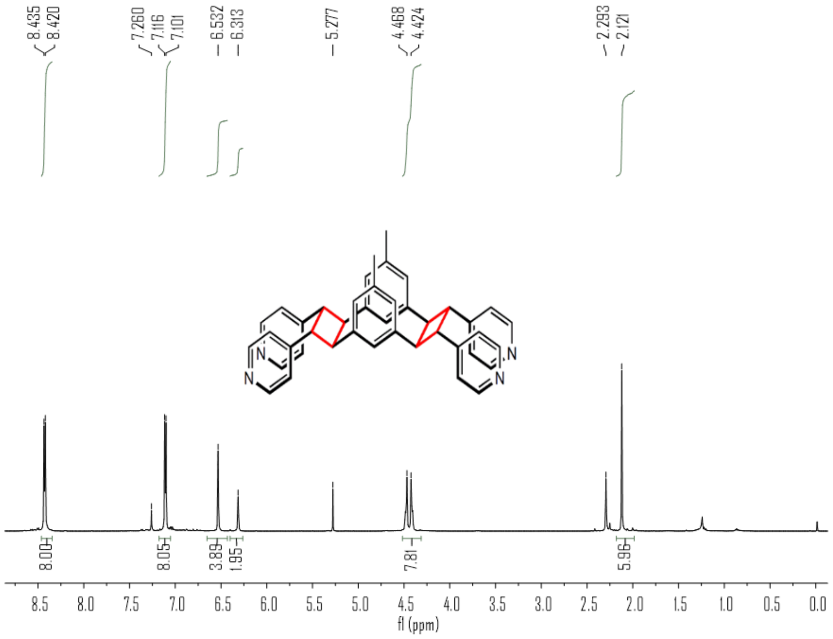

(a)

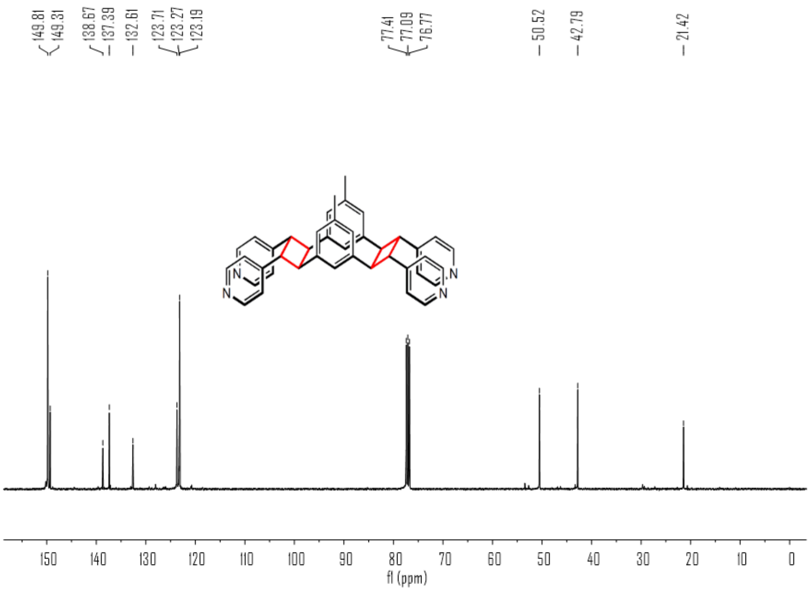
149.81
149.31
138.67
137.39
132.61
123.71
123.27
123.19
77.41
77.09
76.77
50.52
42.79
21.42
N
N
N
N
150
140
130
120
110
100
90
80
70
60
50
40
30
20
10
0
f1 (ppm)

(b)

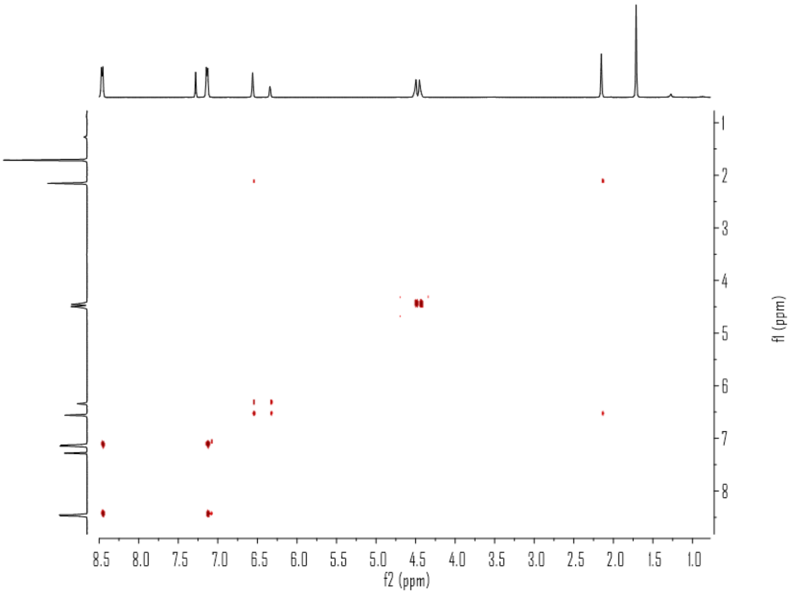
8.5
8.0
7.5
7.0
6.5
6.0
5.5
5.0
4.5
4.0
3.5
3.0
2.5
2.0
1.5
1.0
f2 (ppm)
f1 (ppm)

(c)

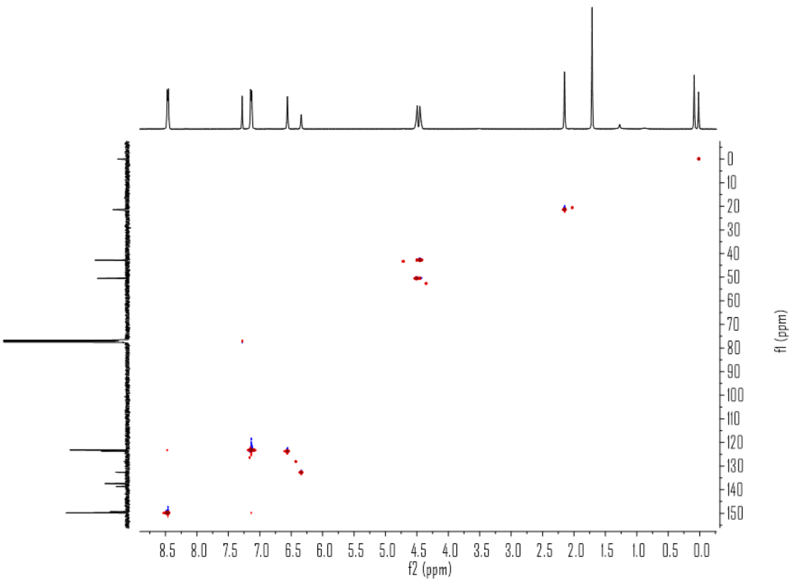

(d)

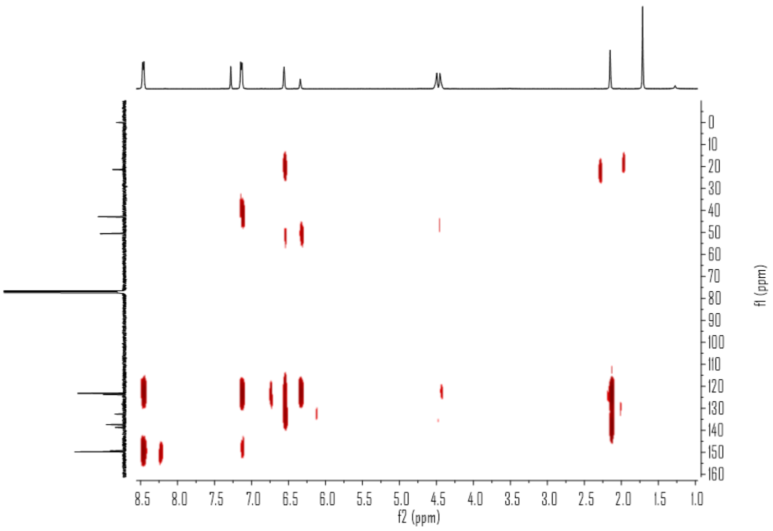

(e)

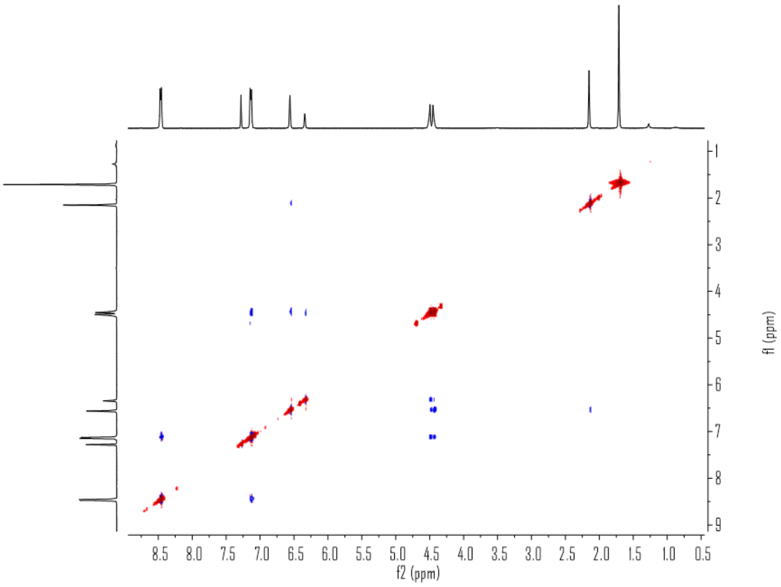

(f)

Figure S2. The ^{1}H (a), ^{13}C (b), H-H COSY (c), HSQC (d), HMBC (e) and NOESY (f) NMR spectra of **Isomer 1α** in $CDCl_3$.

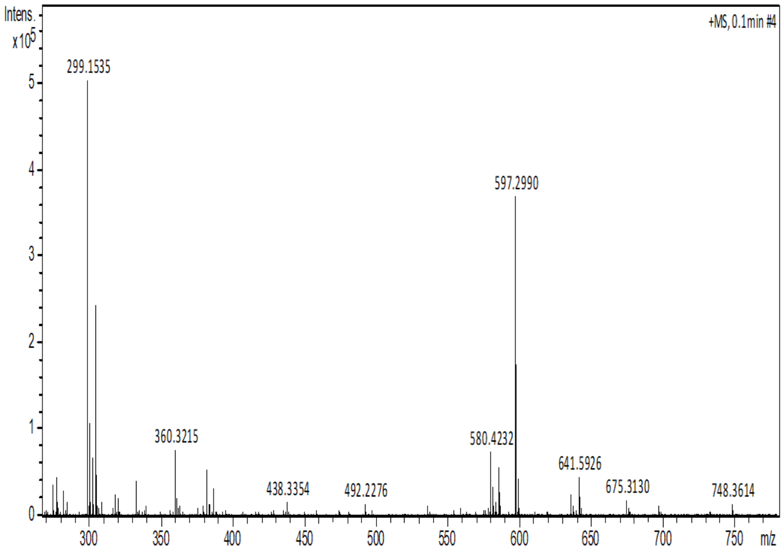

Figure S3. The positive-ion ESI mass spectrum of **Isomer 1α**.

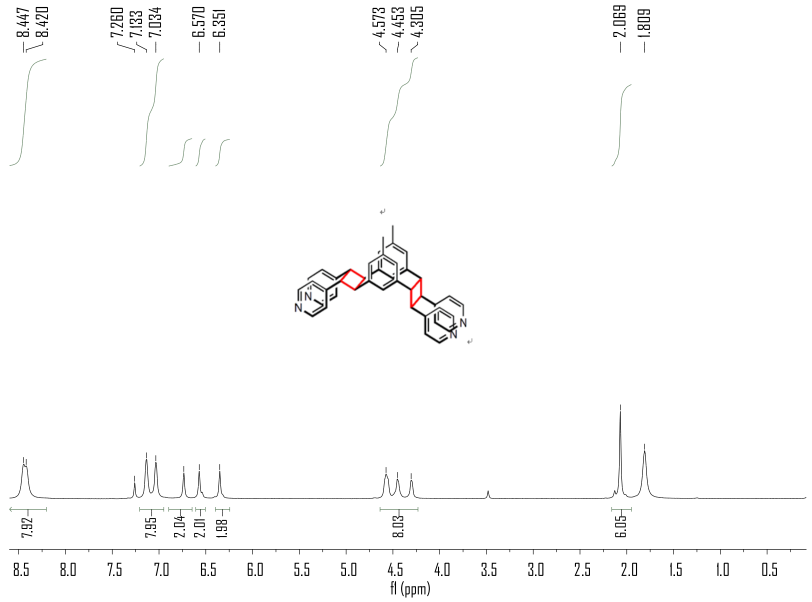
8.447
8.420
7.260
7.133
7.034
6.570
6.351
4.573
4.453
4.305
2.069
1.809
7.92
7.95
2.04
2.01
1.98
8.03
6.05
8.5 8.0 7.5 7.0 6.5 6.0 5.5 5.0 4.5 4.0 3.5 3.0 2.5 2.0 1.5 1.0 0.5
f1 (ppm)

(a)

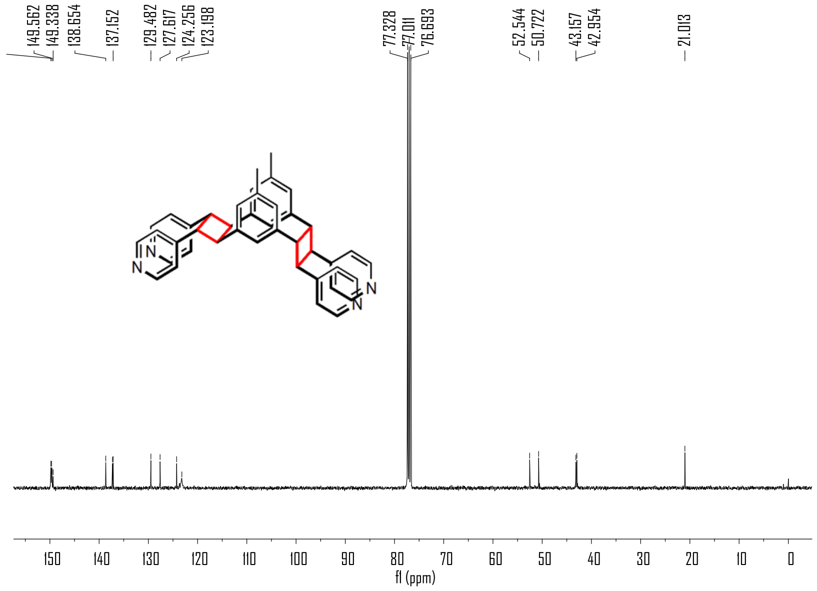
149.562
149.338
138.654
137.152
129.482
127.617
124.256
123.198
77.328
77.011
76.693
52.544
50.722
43.157
42.954
21.013
150 140 130 120 110 100 90 80 70 60 50 40 30 20 10 0
f1 (ppm)

(b)

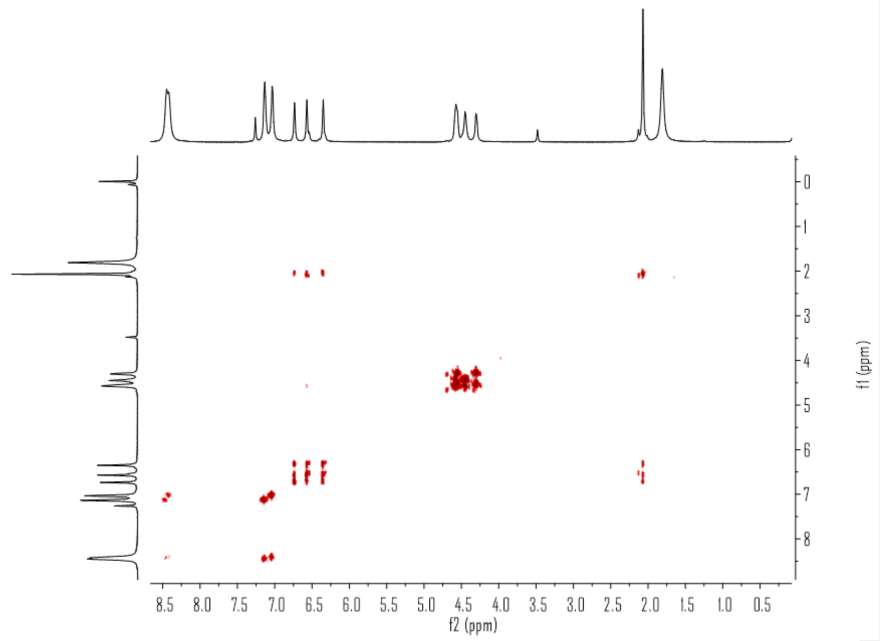

(c)

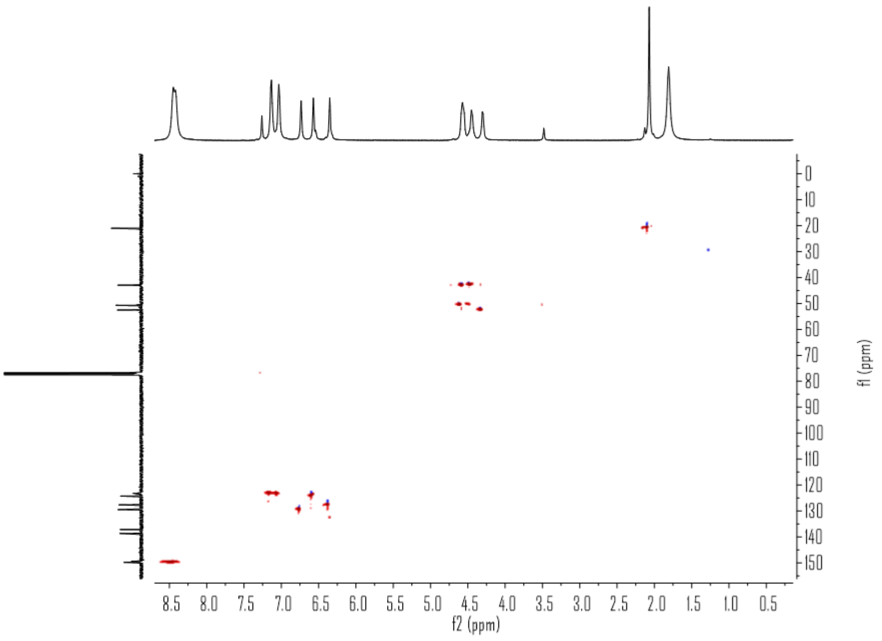

(d)

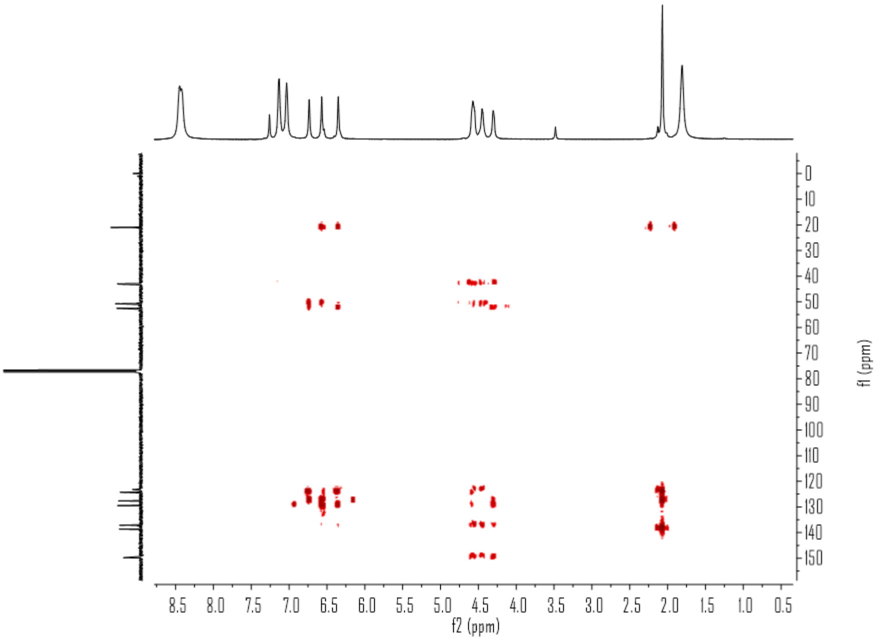

(e)

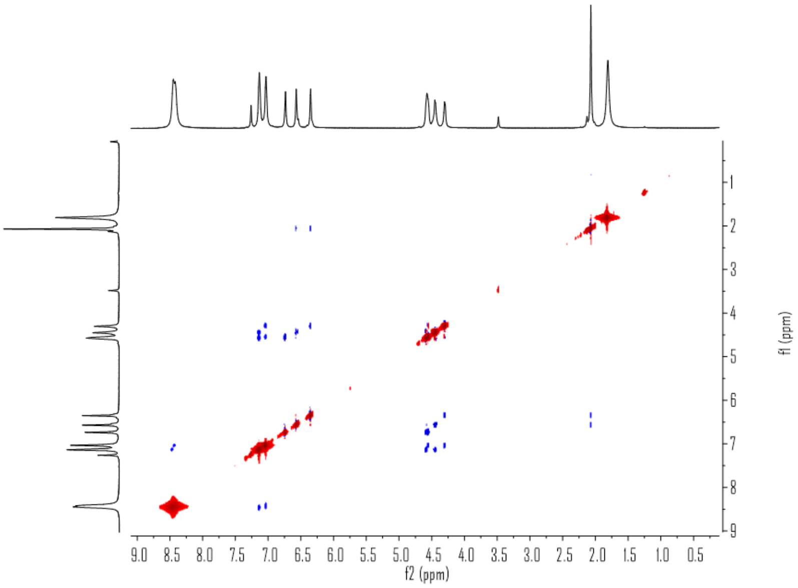

(f)

Figure S4. The ^{1}H (a), ^{13}C (b), H-H COSY (c), HSQC (d), HMBC (e) and NOESY (f) NMR spectra of **Isomer 1β** in $CDCl_3$.

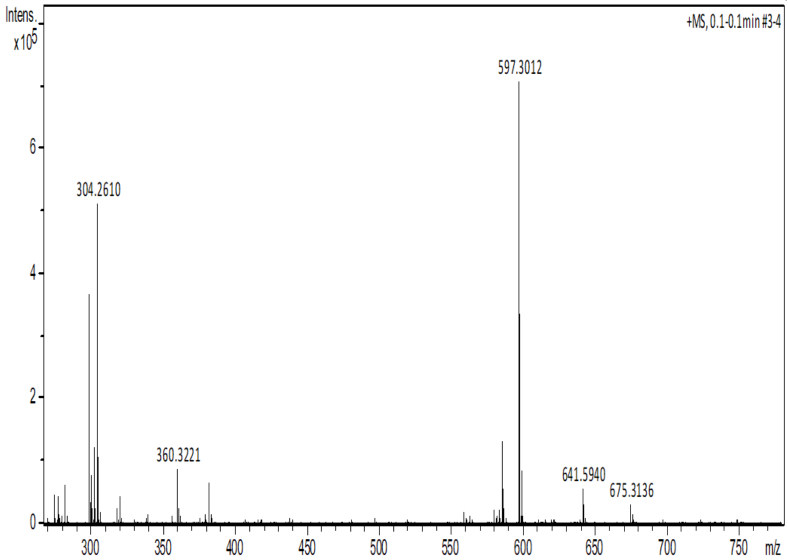

Figure S5. The positive-ion ESI mass spectrum of **Isomer 1β**.

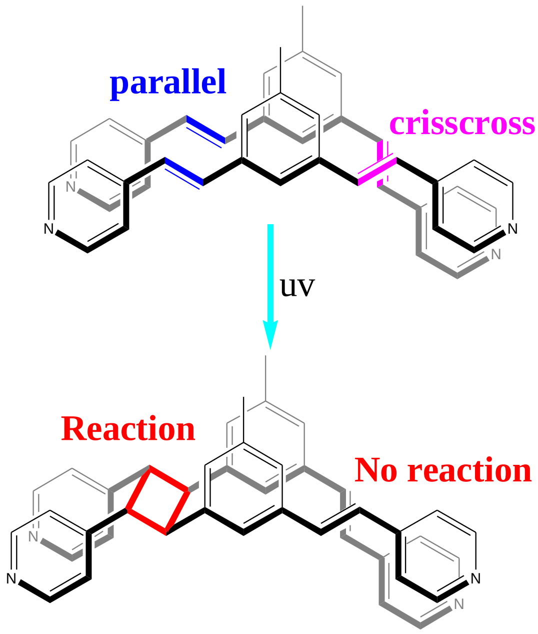

Scheme S2. Photodimerization between the parallel arranged C=C with the crisscross aligned C=C unreacted.

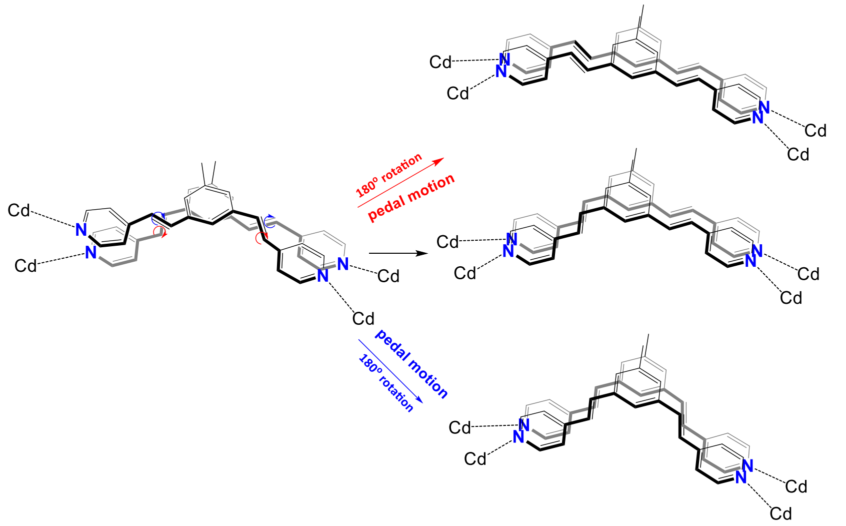

Scheme S3. Representation of the pedal motion in adjacent CH_3-3,5-bpeb molecules.

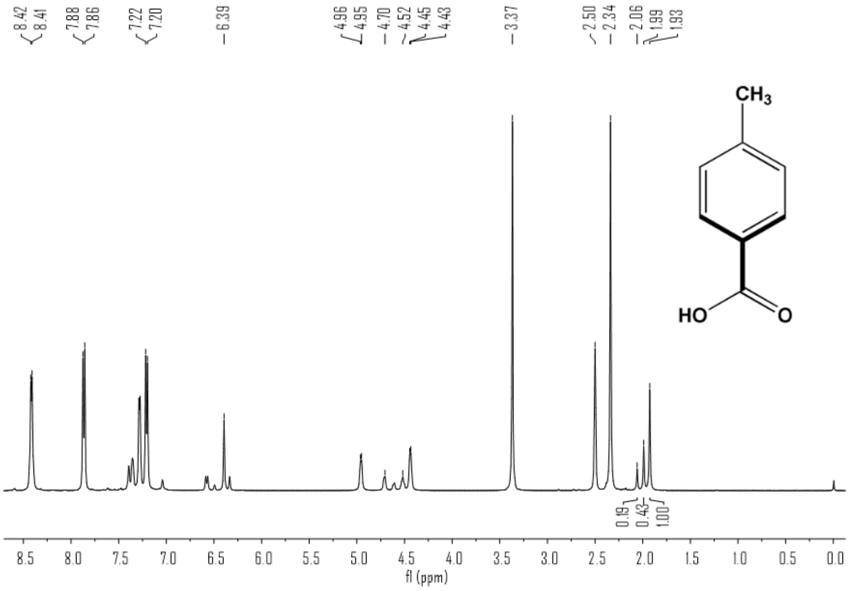

Figure S6. ^{1}H NMR spectrum of **CP$_{3c}$** (d_6-DMSO, after UV irradiation at 373K) .

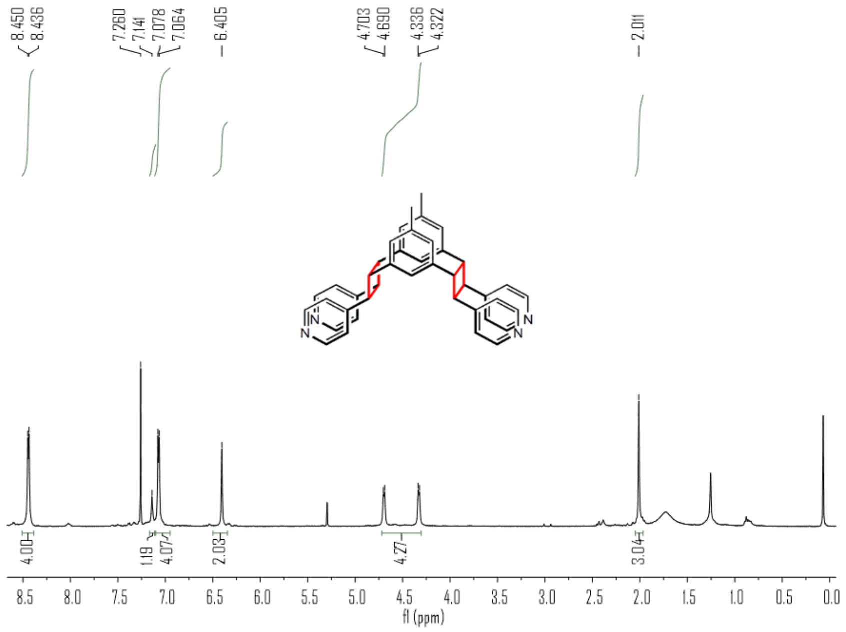
8.450
8.436
7.260
7.141
7.078
7.064
6.405
4.703
4.690
4.336
4.322
2.011
N
N
N
N
4.00
1.19
4.07
2.03
4.27
3.04
8.5 8.0 7.5 7.0 6.5 6.0 5.5 5.0 4.5 4.0 3.5 3.0 2.5 2.0 1.5 1.0 0.5 0.0
f1 (ppm)

(a)

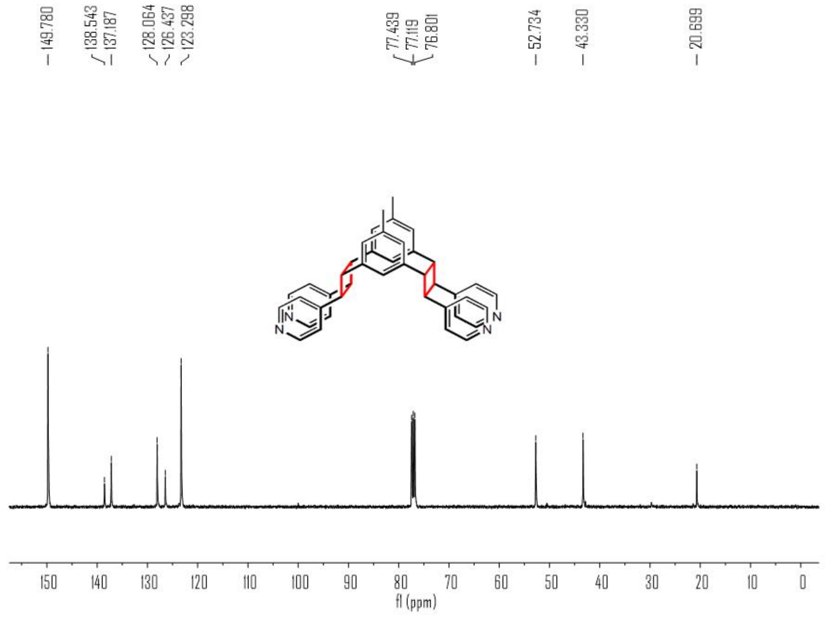
149.780
138.543
137.187
128.064
126.437
123.298
77.439
77.119
76.801
52.734
43.330
20.699
N
N
N
N
150 140 130 120 110 100 90 80 70 60 50 40 30 20 10 0
f1 (ppm)

(b)

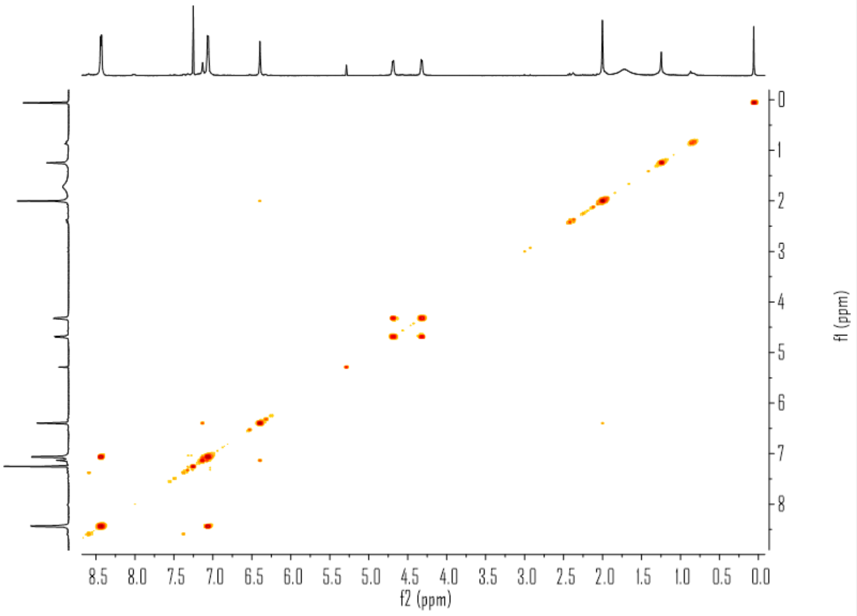

8.5
8.0
7.5
7.0
6.5
6.0
5.5
5.0
4.5
4.0
3.5
3.0
2.5
2.0
1.5
1.0
0.5
0.0
f2 (ppm)
0
1
2
3
4
5
6
7
8
f1 (ppm)

(c)

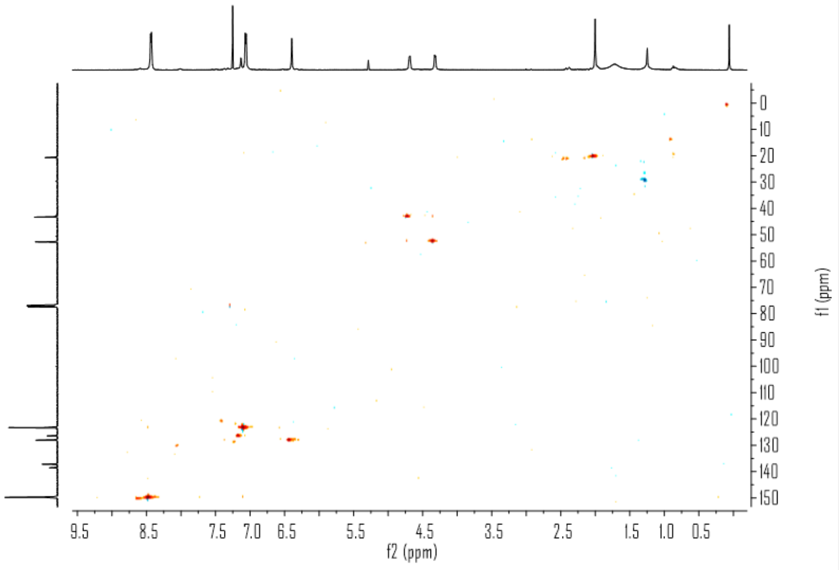

9.5
8.5
7.5
7.0
6.5
5.5
4.5
3.5
2.5
1.5
1.0
0.5
f2 (ppm)
0
10
20
30
40
50
60
70
80
90
100
110
120
130
140
150
f1 (ppm)

(d)

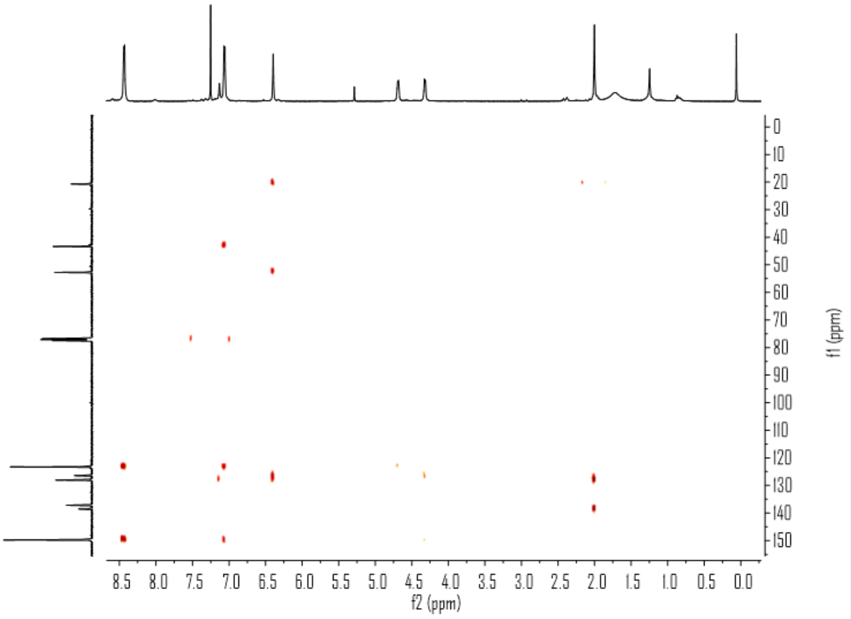

(e)

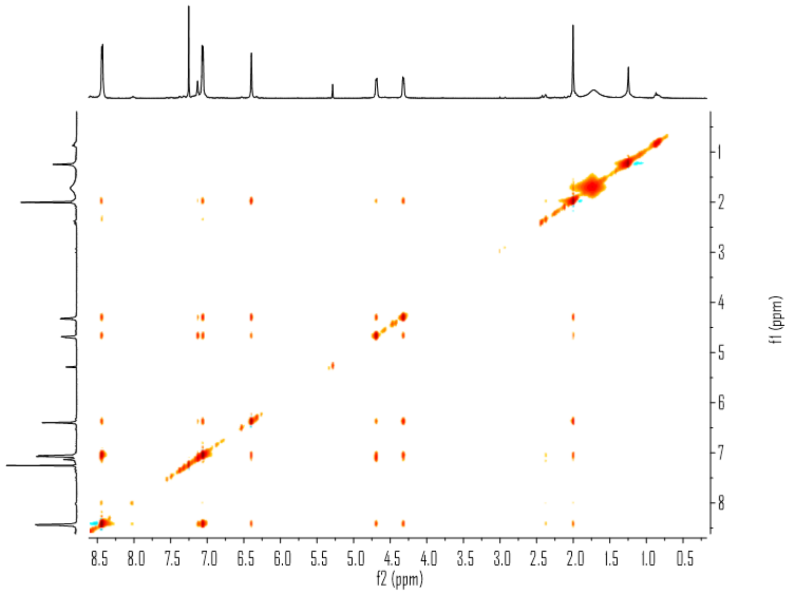

(f)

Figure S7. The ^{1}H (a), ^{13}C (b), H-H COSY (c), HSQC (d), HMBC (e) and NOESY (f) NMR spectra of **Isomer 1γ** in $CDCl_3$.

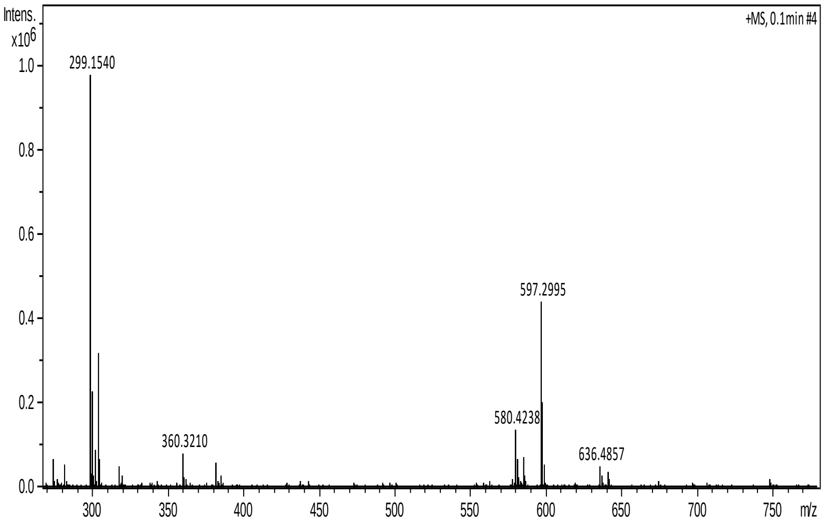

Figure S8. The positive-ion ESI mass spectrum of **Isomer 1γ**.

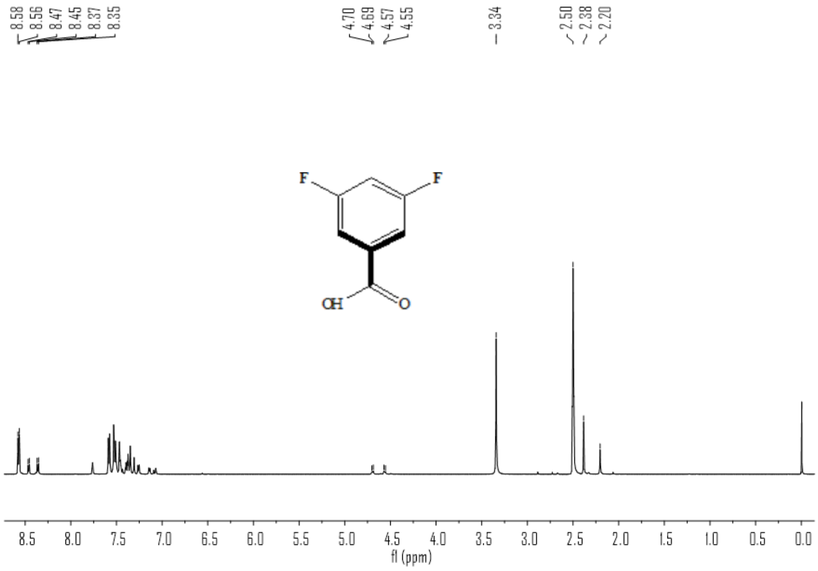

(a)

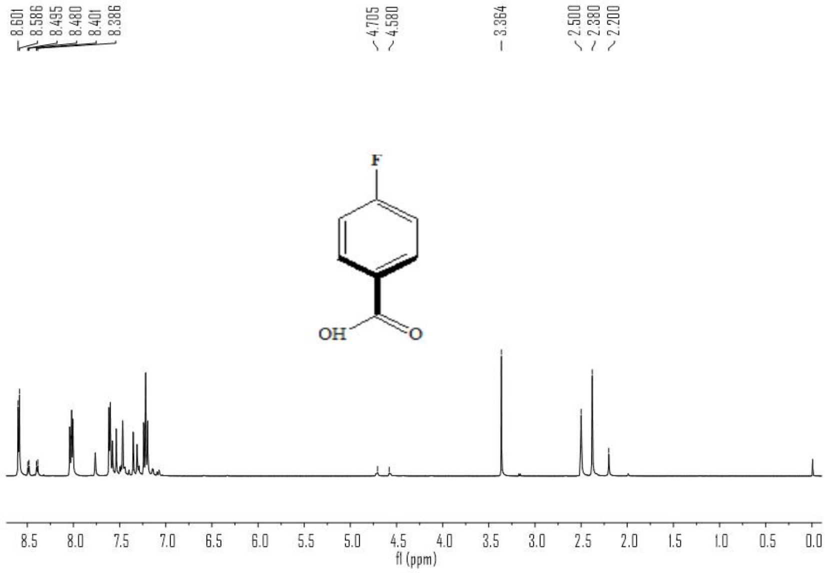

(b)

Figure S9. ^{1}H NMR spectrum **CP3**' (d_6-DMSO **CP3** after irradiation >420 nm).

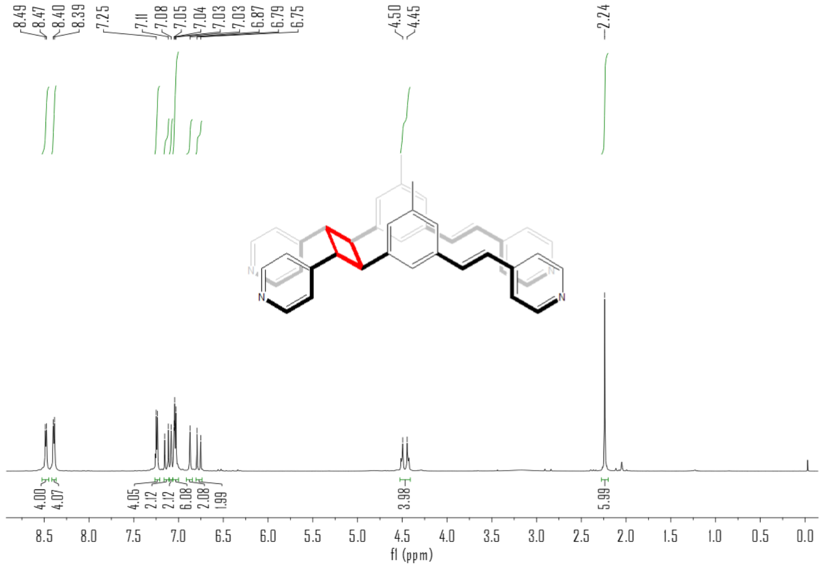

(a)

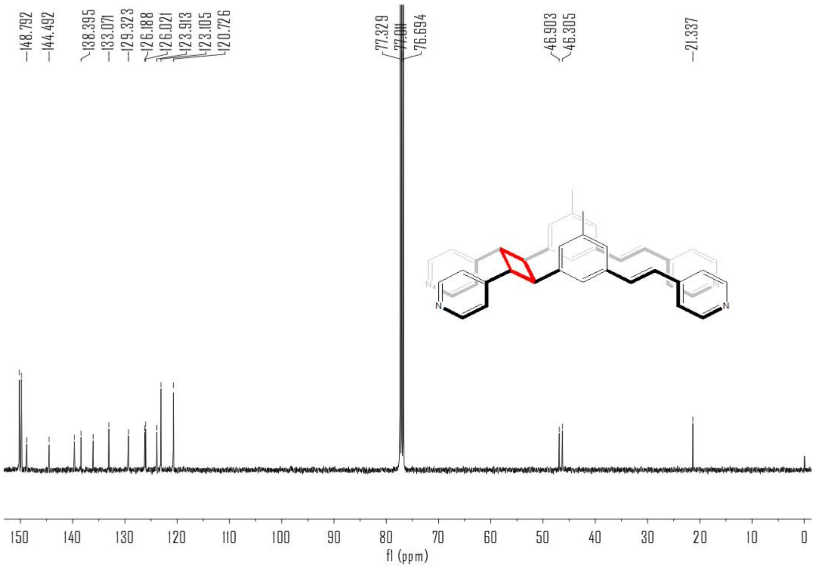

148.792
144.492
138.395
133.071
129.323
126.188
126.021
123.913
123.105
120.726
77.329
77.011
76.694
46.903
46.305
21.337
150 140 130 120 110 100 90 80 70 60 50 40 30 20 10 0
f1 (ppm)

(b)

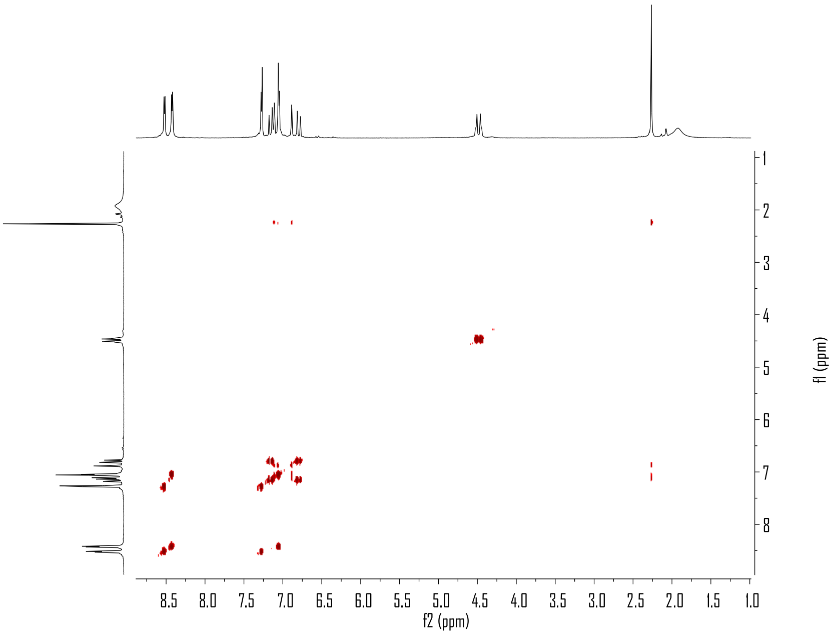

8.5 8.0 7.5 7.0 6.5 6.0 5.5 5.0 4.5 4.0 3.5 3.0 2.5 2.0 1.5 1.0
f2 (ppm)
f1 (ppm)

(c)

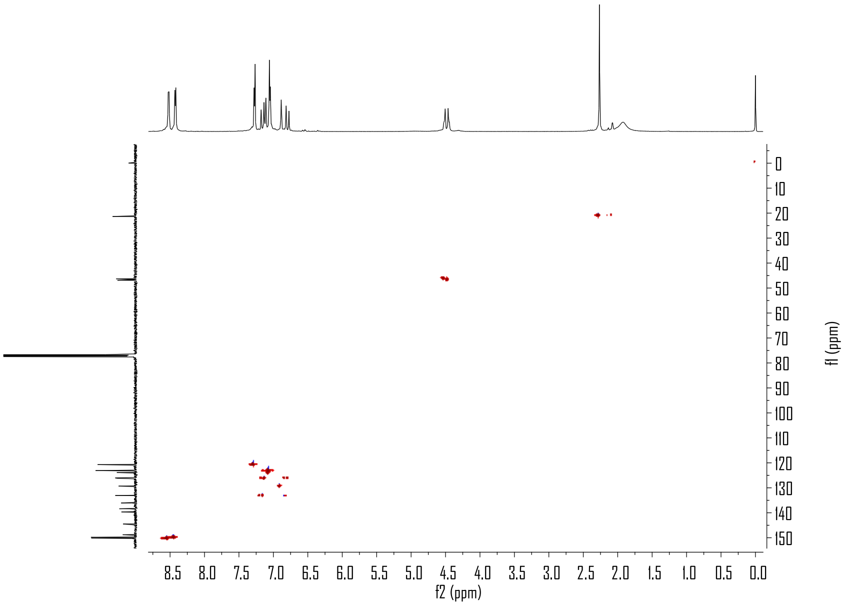

(d)

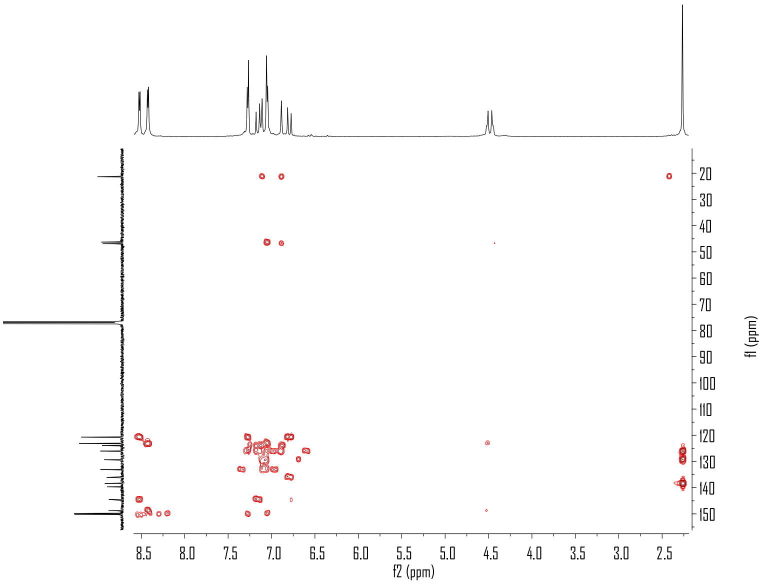

(e)

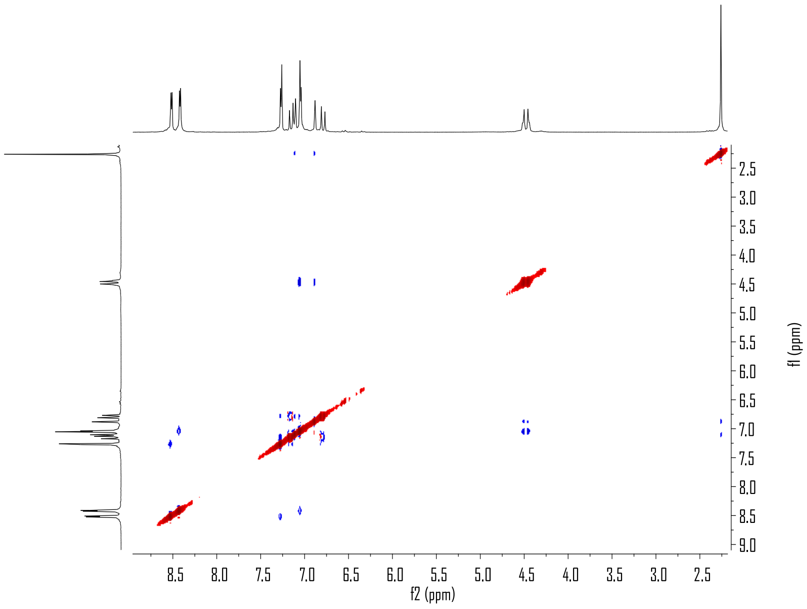

(f)

Figure S10. The ^{1}H (a), ^{13}C (b), H-H COSY (c), HSQC (d), HMBC (e) and NOESY (f) NMR spectra of **Isomer 2** in $CDCl_3$.

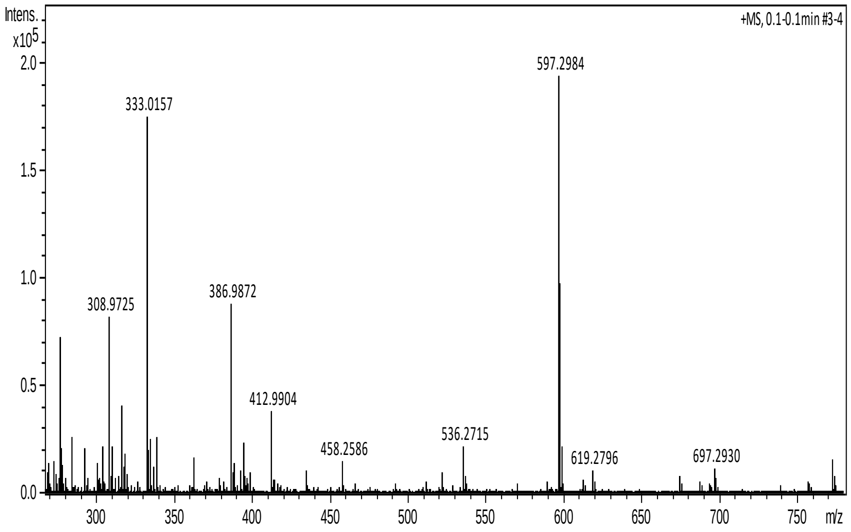

Figure S11. The positive-ion ESI mass spectrum of **Isomer 2**.

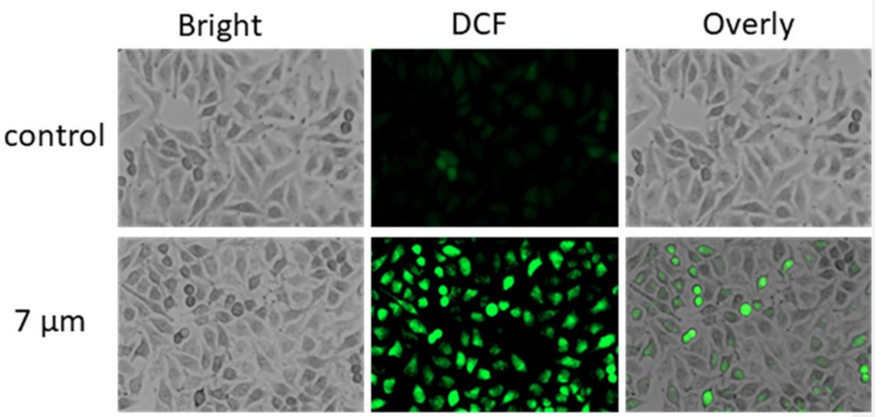

Figure S12. Changes of ROS on T-24 cells treated with **Isomer 2**.

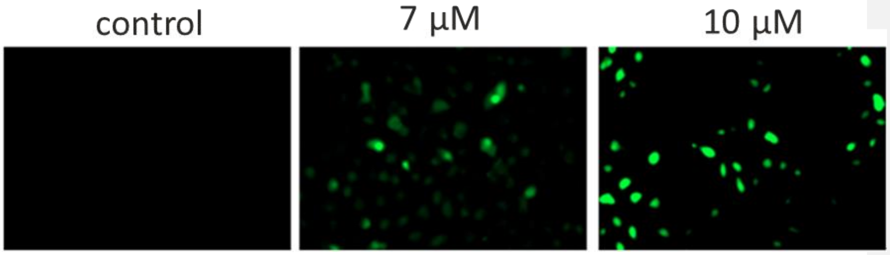

Figure S13. Changes in Ca^{2+} concentration in T-24 cells treated with **Isomer 2**.

References

[1] M.F. Wang, Y. Mi, F.L. Hu, Z. Niu, X.H. Yin, Q. Huang, et al., Coordination-Driven Stereospecific Control Strategy for Pure Cycloisomers in Solid-State Diene Photocycloaddition, J Am Chem Soc, 142(2020) 700-4.
[2] A. C. G. Sheldrick, Sect. C: Struct. Chem., 2015, 71, 3–8
[3] M.X. He, Z.Y. Mo, Z.Q. Wang, S.Y. Cheng, R.R. Xie, H.T. Tang, et al., Electrochemical Synthesis of 1-Naphthols by Intermolecular Annulation of Alkynes with 1,3-Dicarbonyl Compounds, Org Lett, 22(2020) 724-8.
